# Evaluation of the Role of PRP in Acute Tibial Shaft Fractures Fixation With IM Nail: A Double-Blinded Randomized Controlled Trial

**DOI:** 10.1155/aort/5642601

**Published:** 2025-04-07

**Authors:** Wittawat Boonyanuwat, BhupaAk Engkapawastr, Pinkawas Kongmalai

**Affiliations:** ^1^Department of Orthopaedics, Faculty of Medicine, Srinakharinwirot University, 62 Moo 7, Rangsit-Nakhon Nayok Road, Ongkharak, Nakhon Nayok 26120, Thailand; ^2^Department of Orthopedics, Bang Lamung Hospital, 699 Moo 5, Muang Pattaya, Bang Lamung District, Chon Buri 20150, Thailand; ^3^Department of Orthopedics, Faculty of Medicine, Kasetsart University, 50 Ngamwongwan Road, Lat Yao, Chatuchak, Bangkok 10900, Thailand

**Keywords:** AO 42-C3, callus formation, fracture healing, intramedullary nailing, platelet-rich plasma, tibial shaft fractures

## Abstract

**Introduction:** Tibial shaft fractures, particularly those classified as AO 42-C3, represent a significant orthopedic challenge due to their high risk of delayed union or nonunion. Intramedullary nailing is a widely used treatment, though optimizing biological healing remains essential. Platelet-rich plasma (PRP), containing abundant growth factors, has been suggested as a therapeutic aid to enhance fracture healing.

**Methods:** A randomized controlled trial was conducted, including 32 patients diagnosed with acute AO 42-C3 pure diaphyseal tibial fractures. Patients were randomized into two groups: 16 received PRP injections, while 16 received normal saline solution (NSS) injections as a control. The primary outcome, cortex-to-callus ratio, was assessed via radiographs over a 6-month follow-up. Secondary outcomes included patient-reported measures such as the SF-36 and EQ-5D-3L questionnaires and time to union.

**Results:** The PRP group exhibited a significantly higher cortex-to-callus ratio during the third and fourth months of follow-up (*p* < 0.05), indicating accelerated callus formation. Moreover, the PRP group exhibited a statistically significant reduction in time to union compared with the NSS group (*p* < 0.05). Although other patient-reported outcomes did not show significant differences, the PRP group displayed an upward trend in SF-36 scores (*p* < 0.05).

**Discussion:** PRP significantly enhances midstage fracture healing in tibial shaft fractures, as evidenced by improved callus formation and reduced time to union. These results indicate that PRP holds promise as a therapeutic adjunct for managing tibial fractures. Additional studies with larger sample sizes and diverse fixation techniques are needed to validate these findings and further assess the broader potential of PRP in orthopedic practice.

**Summary**

• PRP enhances midstage fracture healing in acute tibial shaft fractures, improving callus formation and reducing time to union.
• The study demonstrates the efficacy of PRP as a biological adjunct in tibial fractures treated with intramedullary nailing.
• Findings suggest that PRP accelerates fracture recovery, offering a promising therapeutic option in orthopedic trauma care.

## 1. Introduction

Tibial shaft fractures represent a common orthopedic challenge, with an annual incidence rate of 16.9 per 100,000 individuals [1]. Because of the subcutaneous boundary conditions on the anteromedial side, the tibia has a poorer blood supply, which elevates the risk of delay union and nonunion [2, 3]. To prevent nonunion, a complex interplay between biomechanical and biological factors is crucial. Intramedullary nailing is considered a reliable option for diaphyseal tibial fractures due to its many biomechanical advantages. It ensures predictable bone realignment, rapid healing, early limb functionality, and the preservation of soft tissue critical for the bone healing process [4]. Meanwhile, the biological aspect of healing encompasses various factors, including comorbidities such as smoking, diabetes, infections, and the influence of specific medications, all of which can significantly impact the bone healing processes.

Biological augmentation plays a pivotal role in enhancing bone healing processes. As a form of regenerative medicine, platelet-rich plasma (PRP) is gaining recognition for its ability to enhance the early stages of the healing process by delivering a biological boost. Modifying the local fracture environment by applying growth factors is gaining recognition as a new treatment approach, designed to lower the risk of nonunion or delayed healing in bone fractures [5–7].

While there have been studies reporting the use of PRP in tibial nonunion [8, 9], prospective clinical studies of PRP in acute tibial shaft fractures are scarce [10]. Our aim is to evaluate the role of PRP by assessing clinical and radiological outcomes in patients undergoing intramedullary nailing for tibial shaft fractures. We hypothesize that PRP may enhance fracture healing in acute diaphyseal closed tibial fractures by supplying a hematoma and various growth factors.

## 2. Materials and Methods

### 2.1. Study Design and Setting

This randomized controlled trial was conducted at the Princess Maha Chakri Medical Center, a tertiary referral care center affiliated with the Faculty of Medicine at Srinakharinwirot University. All participants provided informed consent before enrolling in the study. The local ethics committee (SWUEC345/61) approved the study, which was carried out in compliance with the ethical principles of the 1964 Declaration of Helsinki and its 2000 revision.

### 2.2. Participants

Between January 2018 and December 2020, a total of 35 patients with a confirmed diagnosis of acute diaphyseal tibial fracture were considered potentially eligible. Inclusion criteria included individuals aged between 18 and 60 years who consented to undergo surgery with intramedullary nail fixation, were diagnosed with a highly comminuted fracture pattern classified as AO 42-C3 pure diaphyseal fractures, had no life-threatening conditions, had either not smoked, or had ceased smoking for at least 3 months, had no underlying diseases that would inhibit bone healing, did not use drugs that affect calcium regulation, and had a minimum follow-up period of 6 months after surgery. Exclusion criteria included patients with open diaphyseal fractures, traumatic events in the same area requiring reoperation, those unsuitable for autologous donation (platelet count < 130 × 10^9^/L), individuals with thrombocytopenia, and those who declined to provide consent.

**Figure 1:**
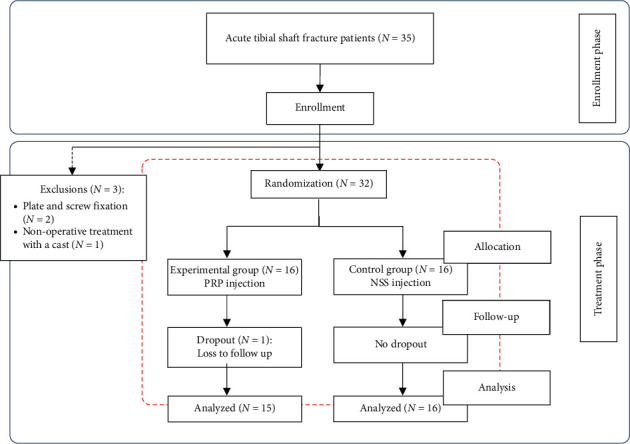
CONSORT flow diagram.

Based on these criteria, two patients were excluded due to a change in the treatment plan to plate and screw fixation, and one patient opted for conservative treatment with a cast. Therefore, 32 patients were randomized into two groups: 16 patients received PRP injection (PRP group) and 16 patients received NSS injection (NSS group). One patient in the PRP group was lost to follow-up. Ultimately, there were 15 patients in the PRP group and 16 patients in the NSS group. Randomization was performed using a computer generated sequence of random numbers and was assigned on the day of surgery. The flow diagram of randomized patients is illustrated in Figure 1. The intention-to-treat analysis method was employed in this study.

### 2.3. Preparation of PRP

On the day of surgery, PRP was prepared under sterile conditions in the Department of Blood Transfusion, using the patient's own blood and processed with a blood bag centrifuge (SORVALL RC 3BP; Thermo Fisher Scientific). Blood was taken from all patients to maintain consistency and blinding. This ensured that all patients underwent the same procedural steps, regardless of whether they were in the PRP or NSS group. The collection of blood from the NSS group was necessary to prevent unblinding of the treating surgeon, thereby eliminating potential bias in postoperative assessments.

A total of 70 mL of blood was drawn from the antecubital vein using a closed sterile technique, and it was collected into a blood bag. The volume collected (70 mL) is within the safe range for adult patients and significantly lower than standard blood donation, posing minimal risk to participants. No adverse effects related to blood sampling were observed. To prevent clotting, citrate phosphate dextrose adenine was added to the blood bag at a ratio of 1:9. After a 10 min centrifugation at 2000 rpm, the blood separated into three basic components: red blood cells settled at the lowest level, platelets in the middle, and platelet-poor plasma (PPP) at the top.

**Figure 2:**
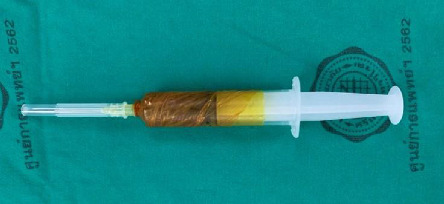
The final step of PRP preparation involved collecting 5 mL of PRP into a specialized 3M Ioband-wrapped syringe to ensure blinding. The opaque covering prevented visual identification of the contents, maintaining the double-blinded design of the study before injection at the fracture site.

The PRP layer was transferred to a second blood bag using a closed system and centrifuged again at 2800 rpm for 10 min. This step separated the blood into two layers: the supernatant, which became PPP, and the lower layer containing PRP. After separating the PPP, the PRP was activated by adding autologous thrombin (0.2 mL per mL of PRP), followed by calcium gluconate (0.2 mL per mL of PRP) [11]. Finally, 5 mL of PRP was drawn into a syringe. The preparation of PRP and saline injections was done in a separate room by a team member not involved in the surgical procedure. This team member then wrapped the syringes in an opaque covering before bringing them to the operating room to ensure that the surgeon could not see the color of the contents, thus maintaining blinding for the surgeon (Figure 2).

### 2.4. Intervention

Preoperative antibiotic prophylaxis was administered to all patients. Following the induction of spinal anesthesia, surgical intramedullary nailing fixation was performed by a single fellowship-trained orthopedic trauma surgeon. All surgical procedures were performed using a standard infrapatellar approach with reamed intramedullary nailing. The Tibial Intramedullary Nail Instruments (Changzhou Waston Medical Appliance Co., China) was used in all cases, with a diameter ranging from 8 to 10 mm, selected based on patient anatomy. After the wound closure in the operating room, a 5 mL injection of either PRP or NSS was administered at the fracture site under fluoroscopic guidance using a sterile technique. During the injection, the surgeon focused on fluoroscopic guidance rather than the syringe itself. In addition, the injection needle was immediately removed and disposed of in a sharps container after administration to prevent visual identification of the syringe contents. This protocol, including the NSS injection in the control group, ensured that both the surgeon and patients remained unaware of the injected substance. It was performed to maintain blinding and procedural consistency, preventing potential bias.

### 2.5. Descriptive Data

There were no significant differences between the groups regarding age, gender, affected body part, or comorbidities. However, it is notable that the mean BMI ± SD was lower in the PRP group (23.47 ± 2.60) compared with the NSS group (26.63 ± 5.36) (Table 1).

### 2.6. Postoperative Rehabilitation

Patients received guidance on appropriate passive and active exercises as part of an early rehabilitation protocol. Suture removal took place during the initial follow-up appointment at 2 weeks postsurgery. Subsequently, patients underwent monthly follow-up assessments. The final evaluation was conducted at the 6-month milestone.

### 2.7. Primary and Secondary Study Outcomes

**Figure 3:**
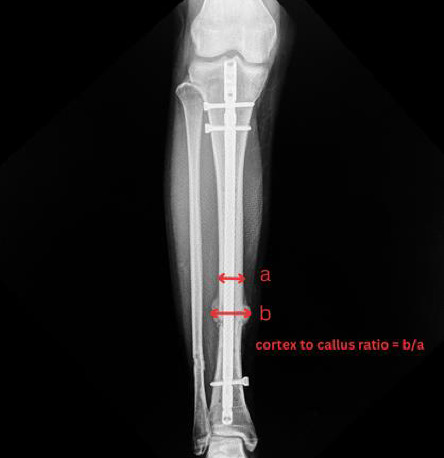
Radiographic measurement of the cortex-to-callus ratio. The cortical bone thickness (a) and total callus thickness (b) were measured at the fracture site. The cortex-to-callus ratio was calculated as b/a, representing the proportion of callus formation relative to the original cortex.

The primary outcome was the cortex-to-callus ratio, assessed using previously established methods [12]. It was calculated by dividing the total callus thickness by the cortical bone thickness at the fracture site, as assessed on radiographs (Figure 3). Radiological union was defined as the presence of a bridging callus in at least three of four cortices on both AP and lateral radiographs. For the secondary outcome, it included a satisfactory score, assessed using the Short Form-36 (SF-36) questionnaire, and a functional score, assessed using the EQ-5D-3L questionnaire.

### 2.8. Statistical Analysis

The sample size calculation was performed using Stata Version 13.0 (StataCorp, 2013), based on the formula for repeated measures data developed by Frison and Pocock [13]. The mean and standard deviation of the cortex-to-callus ratio were derived from a previously published study, where the PRP group had a mean and standard deviation of 1.50 ± 0.45 mm, while the control group had 1.30 ± 0.55 mm [11]. The formula suggested a sample size of 15 individuals per group, which is considered sufficiently to detect clinically meaningful differences with a statistical power of 82.20% at a significance level of 5%. We expected a dropout rate or noncompliance rate within the study of 10%. Therefore, each group required a sample size of 16 individuals. Consequently, the total sample size for this research project was 32 individuals.

Baseline demographic data for study participants were collected and analyzed using suitable statistical methods, including the independent *t*-test and chi-square test. Statistical significance was set at a *p* value of less than 0.05. For the assessment of the cortex-to-callus ratio during the 6-month follow-up period, we calculated the mean difference at baseline using the independent *t*-test. In addition, we determined the mean difference at 2–6 months while adjusting for baseline measurements and all relevant confounding factors using linear regression. The overall mean difference was calculated using generalized estimating equations implemented under the generalized linear model. We assessed patient-reported outcome measures and the time to union using the independent *t*-test and the Mann–Whitney test. A *p* value of less than 0.05 was considered statistically significant.

## 3. Results

The mean time from injury to surgical intervention was 9 days. The mean cortex-to-callus ratio during the 6-month follow-up period did not exhibit a statistically significant difference between the PRP and NSS groups in the first, second, fifth, and sixth months. However, there was a statistically significant increase in the mean cortex-to-callus ratio in the PRP group compared with the NSS group in the third and fourth months (*p* < 0.05) (Table 2).

No significant differences were observed in patient-reported outcome measurements, including VAS pain and EQ-5D-3L, between the two groups. Nevertheless, an increasing trend in SF-36 scores was noted in the PRP group compared with the NSS group, with a statistically significant difference between the two groups (*p* < 0.05) (Table 3).

The mean time to union decreased in the PRP group compared with the NSS group, and a statistically significant difference was observed (*p* < 0.05) (Table 4).

## 4. Discussion

Tibial shaft fractures can lead to substantial short- and long-term complications [2, 14]. Approximately 10%–15% of the fracture patients may experience impaired healing, potentially resulting in nonunion. Therefore, a nonunion tibial fracture may require multiple refixations [15]. In the case of tibial shaft fractures, 1 year after undergoing intramedullary nailing, a significant proportion of patients may develop nonunion [8]. Successful fracture healing requires consideration of both biomechanical and biological factors during treatment. The biological aspect encompasses numerous factors, including smoking, diabetes, vascular diseases, infection, and specific medication usage, all of which can profoundly influence the bone healing processes. PRP is an autologous blood-derived product known for its biological safety and rich content of growth factors that may play a pivotal role in fracture healing and bone regeneration in tibial shaft fractures [16, 17]. A systematic review by Jamal et al. found that PRP can enhance bone healing by accelerating callus formation and reducing time to union, particularly in lower-extremity fractures [7]. However, variability in PRP preparation and limited high-level evidence remain challenges. This highlights the need for rigorous clinical trials, such as our double-blinded randomized controlled study, to evaluate PRP's efficacy in tibial shaft fractures treated with intramedullary nailing.

We found that the mean cortex-to-callus ratio during the 6-month follow-up period did not exhibit a statistically significant difference between the PRP and NSS groups in the first, second, fifth, and sixth months. A statistically significant increase in the mean cortex-to-callus ratio was observed in the PRP group compared with the NSS group during the third and fourth months. This indicates that PRP may notably accelerate fracture healing, especially in the midrecovery stage.

**Figure 4:**
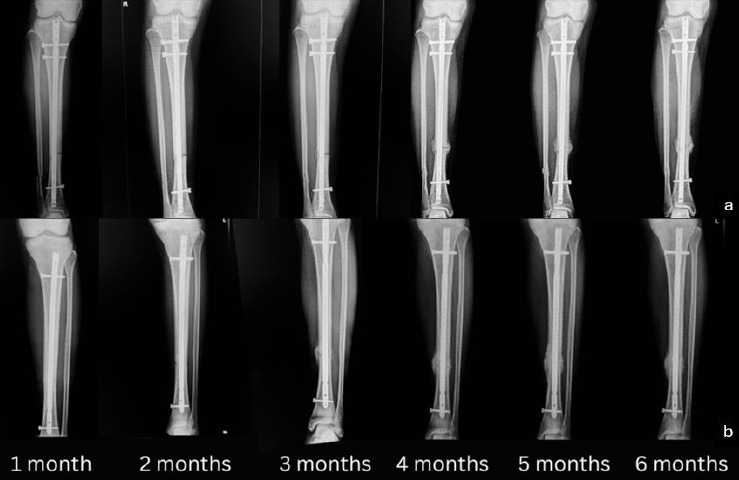
Serial radiographs demonstrate the radiological evolution of callus formation over 6 months in two representative patients from each group. (a) Control group (NSS injection) and (b) PRP group, with monthly follow-ups from 1 to 6 months postoperatively. Callus formation appears more prominent in the PRP group, particularly in the third and fourth months, aligning with the statistically significant increase in the mean cortex-to-callus ratio observed during this period.

The cortex-to-callus ratio is a measure of the relative thickness of the callus compared to the cortical bone at the fracture site. A higher ratio indicates more robust callus formation, which is an essential part of the fracture healing process. The observed increase in the cortex-to-callus ratio in the PRP group during the third and fourth months (Figure 4) may be attributed to several mechanisms. The presence of growth factors such as PDGF, TGF-β, and VEGF in PRP enhances cellular proliferation, differentiation, and angiogenesis. These growth factors are crucial during the midstage of recovery, typically around the third and fourth months, when the initial inflammatory phase has subsided, and the reparative phase is actively progressing.

Our results align with those reported by Mohammed et al., who conducted a study involving patients with midshaft tibial fractures fixed with an IM nail [10]. The patients were split into two groups: one group of 12 received PRP injections, while the other 12 did not. Results showed that those who received the PRP injections experienced significantly faster healing, greater pain reduction, and quicker return to normal function compared to the control group. We believe that our study is superior in two aspects. First, we employed several approaches to ensure a double-blind methodology. Blood was taken from all patients to ensure consistency and that all patients underwent the same procedure, regardless of whether they were in the PRP or NSS group. The preparation of PRP and saline injections was conducted in a separate room by a team member not involved in the surgical procedure. This team member then wrapped the syringes in an opaque covering before bringing them to the operating room, ensuring that the surgeon could not see the color of the contents. In addition, we administered NSS into the fracture site of the control group. By doing so, we ensured that neither the surgeons nor the patients were aware of the treatment allocation. Second, while their results only analyzed patients up to 3 months for all outcomes, we followed patients for a duration of 6 months, which we consider a crucial time point in fracture management. The noteworthy findings in this study pertain to the time to union, which showed a statistically significant decrease in the PRP group compared with the NSS group (*p* < 0.05). These results likely highlight the benefits of a fibrin clot rich in growth factors at the fracture site, enhancing its capacity to act as a scaffold for cell attachment and supporting the mineralization process.

Unlike our findings, which suggest benefits of PRP, Singh et al. reported no significant impact of PRP on the healing process of femoral shaft fractures treated with closed intramedullary nailing. This contrast highlights the variability in PRP's effectiveness depending on fracture type and treatment method [11]. We attribute these discrepancies in outcomes to the distinct nature of the bone fractures. In femoral shaft fractures, nonunion often results from issues such as bone loss or fixation failure. In contrast, tibial fractures are characterized by a paucity of surrounding soft tissues and reduced vascular supply, which are associated with a higher nonunion rate. Because of these challenging conditions, tibial shaft fractures appear to derive substantial benefits from PRP treatment. The enhanced delivery of growth factors and improved angiogenesis provided by PRP can significantly aid the healing process in tibial fractures, making PRP particularly beneficial for these types of fractures compared to femoral fractures.

According to patient-reported outcome measures, Manco et al. studied the arthroscopic treatment of osteochondral lesions in the knee joint [18]. They compared microfracture treatment to microfracture treatment plus PRP application. VAS score, Short Form Health Survey, and the International Knee Documentation Committee Subjective Knee Form were collected as PROMs. All evaluations demonstrated that the combination of microfracture treatment and PRP injection led to superior clinical and functional outcomes in the short-term follow-up compared with other methods. In our study, PRP injection groups also had a statistically significant higher SF-36 score compared with the NSS group (*p* < 0.05).

With a simple technique for preparation, the utilization of PRP derived from a patient's own blood enhances biological safety, making autologous PRP readily available in most healthcare settings for stimulating tissue healing and bone regeneration. Furthermore, the absence of infection complications in both the PRP and control groups underscores the safety profile of PRP treatment.

This study has some limitations. First, the sample size is relatively small. Despite conducting a power analysis to estimate our required sample size, our cohort size remained limited due to the lower incidence of traffic accidents during the government lockdown policy implemented in response to the COVID-19 pandemic. Future studies should focus on incorporating larger sample sizes to enhance statistical power and increase the generalizability of the results. Second, all patients in our cohort underwent fixation with an IM nail, which may introduce potential biological augmentation effects after reaming. This should be kept in mind for further investigation, and additional studies should possibly categorize subjects into groups involving different internal fixation methods, such as traditional open plates and screws or minimally invasive plate osteosynthesis.

## 5. Conclusion

Our study demonstrated that PRP significantly enhances the midstage fracture healing process in tibial shaft fractures, as evidenced by the increased mean cortex-to-callus ratio in the third and fourth months and the reduced time to union. These findings suggest that PRP can effectively improve callus formation and accelerate overall healing in tibial fractures. While the study's double-blinded methodology enhances the reliability of these results, future research with larger sample sizes and diverse fixation methods is needed to further validate and expand upon these findings. PRP presents a promising therapeutic option for improving fracture healing outcomes.

## Figures and Tables

**Table 1:** Baseline demographics of patients in the study.

Characteristics	NSS (*N* = 16)	PRP (*N* = 15)	*p* value
Age (years), mean ± SD	33.19 ± 12.34	38.13 ± 13.36	0.29^∗^
Gender			
Male	9	11	0.32^∗∗^
Female	7	4	
Body mass index (kg/m^2^)	26.63 ± 5.36	23.47 ± 2.60	< 0.05^∗^
Affected part (side, left/right)	9/7	9/6	0.83^∗∗^
Underlying disease			
Hypertension	1	3	—
Diabetes mellitus	0	3	—
Malnutrition	0	0	—
Liver disease	1	0	—
Renal disease	0	0	—
Alcoholic consumption	3	3	—
Smoking	5	1	—

Abbreviation: SD, standard division.

^∗^Independent *t*-test.

^∗∗^Chi-square test.

**Table 2:** Mean cortex to callus ratio during 6 months of follow-up.

Cortex-to-callus ratio	NSS (*N* = 16)	PRP (*N* = 15)	Mean difference	95% CI	*p* value^†^
	Mean ± SD	Mean ± SD			
1 month	1.0592 ± 0.0242	1.0864 ± 0.0251	0.0182	−0.0162–0.0525	0.30
2 months	1.1602 ± 0.0347	1.2036 ± 0.0346	0.0230	−0.0011–0.0471	0.06
3 months	1.2481 ± 0.0366	1.2972 ± 0.0368	0.0401	0.0103–0.0700	< 0.05
4 months	1.3226 ± 0.0278	1.3542 ± 0.0286	0.0236	0.0009–0.0463	< 0.05
5 months	1.3824 ± 0.0280	1.3934 ± 0.0189	0.0031	−0.0158–0.0219	0.74
6 months	1.4235 ± 0.0231	1.4235 ± 0.0187	0.0010	−0.0161–0.0182	0.90
Overall	—	—	0.0182	−0.0030–0.0394	0.09^‡^

Abbreviations: 95% CI, 95% confidence interval; SD, standard division.

^†^The mean difference at baseline using independent *t*-test as well as the mean difference at 2–6 months adjusted for baseline measurements and all confounding factors for each visit using linear regression.

^‡^Overall mean difference using generalized estimating equations (GEEs) implemented under generalized linear model.

**Table 3:** Patient-reported outcome measures.

PROMs	NSS (*N* = 16)	PRP (*N* = 15)	Mean difference	95% CI	*p* value
	Mean ± SD	Mean ± SD			
VAS pain	5.56 ± 2.06	5.60 ± 1.59	0.038	−1.40–1.32	0.96^∗^
EQ-5D-3L	1.19 ± 0.91	1.40 ± 1.12	0.212	−0.96–0.54	0.57^∗^
SF-36, median (IQR)	43.50 (42.00–47.50)	52.00 (47.00–59.00)	7.00	4.00–12.00	< 0.05^∗∗^

*Note:* SF-36: the 36-Item Short Form Health Survey.

Abbreviations: 95% CI, 95% confidence interval; IQR, interquartile range; PROMs, patient-reported outcome measures; SD, standard division; VAS Pain, Visual Analog Scale for Pain.

^∗^Independent *t*-test.

^∗∗^Mann–Whitney test.

**Table 4:** Time to union.

	NSS (*N* = 16)	PRP (*N* = 15)	Mean difference	95% CI	*p* value
	Mean ± SD	Mean ± SD			
Union (weeks)	15.38 ± 2.89	12.53 ± 2.97	2.84	0.68–4.99	< 0.05

Abbreviations: 95% CI, 95% confidence interval; SD, standard division.

## Data Availability

The data that support the findings of this study are available on request from the corresponding author. The data are not publicly available due to privacy or ethical restrictions.
